# Improved agreement and diagnostic accuracy of a cuffless 24-h blood pressure measurement device in clinical practice

**DOI:** 10.1038/s41598-020-80905-x

**Published:** 2021-01-13

**Authors:** Thenral Socrates, Philipp Krisai, Annina S. Vischer, Andrea Meienberg, Michael Mayr, Thilo Burkard

**Affiliations:** 1 Medical Outpatient and Hypertension Clinic, ESH Hypertension Centre of Excellence, University Hospital Basel, Petersgraben 4, 4031 Basel, Switzerland; 2 Department of Cardiology, University Hospital Basel, Basel, Switzerland; 3 Cardiovascular Research Institute Basel, University Hospital Basel, Basel, Switzerland

**Keywords:** Cardiology, Medical research, Risk factors

## Abstract

A cuffless blood pressure (BP) device (TestBP) using pulse transit time is in clinical use, but leads to higher BP values compared to a cuff-based 24 h-BP reference device (RefBP). We evaluated the impact of a recent software update on BP results and TestBP’s ability to differentiate between normo- and hypertension. 71 individuals had TestBP (Somnotouch-NIBP) and RefBP measurements simultaneously performed on either arm. TestBP results with software version V1.5 were compared to V1.4 and RefBP. Mean 24 h (± SD) BP for the RefBP, TestBP-V1.4 and TestBP-V1.5 were systolic 134.0 (± 17.3), 140.8 (± 20) and 139.1 (± 20) mmHg, and diastolic 79.3 (± 11.7), 85.8 (± 14.1) and 83.5 (± 13.0) mmHg, respectively (p-values < 0.001). TestBP-V1.5 area under the curve (95% confidence interval) versus RefBP for hypertension detection was 0.92 (0.86; 0.99), 0.94 (0.88; 0.99) and 0.77 (0.66; 0.88) for systolic and 0.92 (0.86; 0.99), 0.92 (0.85; 0.99) and 0.84 (0.74; 0.94) for diastolic 24 h, awake and asleep BP respectively. TestBP-V1.5 detected elevated systolic/diastolic mean 24 h-BP with a 95%/90% sensitivity and 65%/70% specificity. Highest Youden’s Index was systolic 133 (sensitivity 95%/specificity 80%) and diastolic 87 mmHg (sensitivity 81%/specificity 98%). The update improved the agreement to RefBP. TestBP was excellent for detecting 24 h and awake hypertensive BP values but not for asleep BP values.

## Introduction

Hypertension is a global healthcare burden and the most important modifiable risk factor for cardiovascular disease and stroke^1^. Detecting and managing arterial hypertension is a daily task in medicine. However, much controversy exists on how blood pressure (BP) should be measured and how hypertension should be diagnosed and monitored. Different guidelines recommend non-invasive oscillometric, cuff-based, 24 h ambulatory BP pressure monitoring for the accurate diagnosis of arterial hypertension using validated devices^2,3^. In general, patients are not keen on this BP measurement (BPM) method, finding the repeated inflations of the cuff, especially at night, uncomfortable and disruptive. The stimulation caused by the inflations, during sleep, could also cause the readings to be inaccurate due to sympathetic arousal, pressure on anatomical structures, and measurement artefacts caused by movement^4,5^.

Since 2015, a novel cuffless BP device using pulse transit time (PTT) for the beat-to-beat calculation of BP values has been in clinical use. The device was validated according to the ESH International Protocol Revision 2010 for the Validation of Blood Pressure Measuring Devices in Adults (ESH IP 2010)^6^. After initial calibration with a standard cuff-based measurement, this standard validation technique is carried out over an approximate 25-min period in a non-stimulating environment. This setting does not represent the usual circumstances with which the device is used, namely over a 24 h period with normal activities.

In our previous study, we compared the cuffless device (TestBP) to a standard cuff-based device (RefBP). The novelty of this study was that the measurements took place over a 24 h period with both devices simultaneously worn by the participant, capturing BPM during daily activities and sleep. A significant difference between the two devices was detected. In particular, the cuffless device revealed systematically higher systolic and diastolic values, especially during the asleep phase. This precluded that the values of the devices were not directly interchangeable in a clinical context and needs to be taken into account when implementing the cuffless device in a clinical setting^7^.

In 2018, the TestBP device manufacturer (Somnomedics GmbH, Randersacker Germany) updated the software algorithm for better BP calculation. With the present analysis, we aimed to investigate the impact of this software update on device accuracy compared to the RefBP. Furthermore, we evaluated diagnostic accuracy for the detection of hypertensive BP values to give clinical guidance and possibly derive device specific cut-off values against the gold standard (RefBP).

## Experimental section

The study protocol complies with the Declaration of Helsinki, was approved by the local ethics committee, Ethikkommission Nordwest- und Zentralschweiz (Ethics Commission Northwest and Central Switzerland), (EKNZ 2017-00323), registered (NCT 03054688) and externally monitored. Informed consent was obtained from all participants.

### Device details

Somnotouch-NIBP (Somnomedics GmbH, Randersacker, Germany) is a cuffless, non-invasive system which estimates BP based on the PTT technique (TestBP). Allowing for continuous beat-to-beat BP monitoring. Finger photoplethysmography, three ECG leads and a watch-like-device with integrated actigraph are the hardware of the device. Transit time of a pulse wave from the corresponding ECG R-wave to the finger photoplethysmography signal is measured^6^. Systolic and diastolic BP levels are calculated using non-linear modelling incorporating changes of the PTT and its relation to BP. The measurements are derived after an initial single-cuff based calibration measurement on the contralateral upper arm^8^. Increasing pulse wave propagation results in shorter PTT and is associated with higher BP values. Logically, decreasing pulse wave propagation and longer PTT is linked to lower BP values. Arterial wall stiffness and tension influence pulse wave propagation and PTT, both of which effect BP^9,10^. In this study the reference device (RefBP) was the cuff-based Spacelabs 90217A (Spacelabs Healthcare Inc., USA) 24 h BP monitor^11^. In July 2018, Somnomedics completed an update of the software. This update of the Domino light software version 1.4 led to the current version 1.5 (02/07/2018).

### Study center

In April 2015, the cuffless device was introduced at the Medical Outpatient and Hypertension Clinic at the University Hospital Basel. The study team was trained to use the TestBP correctly and at the initiation of the study, the centre had two years of experience and had performed > 500 measurements. Experienced cardiologists (TB, AV) read all TestBP and RefBP measurements.

### Enrolment

Enrolment took place at the Medical Outpatient and Hypertension Clinic at the University Hospital Basel between May and December 2017. The target sample consisted of consecutively recruited participants with an indication for a 24 h BPM as well as healthy volunteers. Distribution over pre-defined BP ranges and pre-specified recruitment numbers were defined prior to the initiation of the study as reported previously^7^. This included at least 15 participants in low (< 135 mmHg), intermediate (≥ 135 and < 150 mmHg) and high (≥ 150 mmHg) mean-awake systolic BP categories measured by the RefBP. BP categorisation for 24 h mean BP currently does not exist therefore the definition of low, intermediate and high categories was espoused from the National Institute of Health and Care Excellence clinical guidelines^12,13^. In addition to the BP category requirements, at least 15 male and 15 female subjects needed to be included. The ability to give informed consent and age ≥ 25 years as proposed by the ESH-IP 2010 were main inclusion criteria^12^. Exclusion criteria consisted of age < 25 years, a systolic BP difference > 10 mmHg between both upper extremities, atrial fibrillation at the time of enrolment and other medical reasons prohibiting BPM on the upper extremities. For evaluation of inter-arm differences, the following procedure took place: After 5 min of rest, the inter-arm BP difference was assessed sequentially with a single measurement on the right and left upper arm, in a sitting, upright position with legs uncrossed and back supported using the validated, cuff based, oscillometric Omron HBP-1300 device^14,15^.

Active recruitment took place until all recruitment categorizes were fulfilled.

### Measurement procedure

Both devices were mounted on the participant in a sitting, upright position with legs uncrossed and back supported. An appropriately sized cuff was placed on the right arm and connected to the RefBP. The TestBP was placed on the left forearm and connected to the photoplethysmograph on the left index finger and the ECG electrodes according to manufacturer’s instructions. The first manually triggered RefBP measurement was taken after 5 min of rest. This measurement was used as a calibration measurement for the TestBP. The RefBP was programmed for measurements every 20 min from 08:00 to 22:00 and every 30 min during the remaining period. Simultaneously, the TestBP recorded beat-to-beat PTT according to the manufacturer’s instructions and standard programming. Participants were given questionnaires to individually record their activities and sleep schedules.

After completion of the 24 h measurement period, the RefBP measurements were analysed using its own standard software (Spacelabs Healthcare Inc, USA). A minimum of 27 BP values were required to deem the RefBP measurement valid. Otherwise, the data set was excluded^16^. The information about time in bed that we obtained from the patient protocol defined the asleep phase. Mean systolic and diastolic 24 h, awake and asleep values were calculated. TestBP measurements were evaluated twice using the incorporated standard software (Domino Light; Somnomedics GmbH). The first analysis (TestBP-V1.4) used the former software version (Domino Light Version V1.4; Somnomedics GmbH) and the second analysis (TestBP-V1.5) used the updated software version (Domino Light V1.5; Somnomedics GmbH). Automatic detection of artefacts during asleep time (feature included in the Somnotouch Domino light software package) were implemented. Only data sets, with < 50% artefact time, were deemed as valid and included in the final analysis. TB and AV chose the ECG lead with the best signal quality.

Standardised questionnaires were used to evaluate personal and medical factors including intake of antihypertensive medication and biometrics.

### Statistical analysis

Distribution of continuous variables was determined using skewness, kurtosis and visual inspection of the histogram. Continuous data were presented as means (± standard deviations) and compared using paired t-tests. Categorical variables were described as counts (percentages) and compared using chi square tests.

Mean absolute BP differences between the RefBP vs. TestBP-V1.4 and RefBP vs. TestBP-V1.5 were obtained by reversing all negative values to positive values before calculation. Intraclass correlation coefficients between RefBP and both software versions (TestBP-V1.4 and TestBP-V1.5) were calculated to evaluate the general reliability index of a test–retest of the two devices. We followed the guidelines described by Koo and Li, with values less than 0.5, between 0.5 and 0.75, between 0.75 and 0.9, and greater than 0.90 indicating poor, moderate, good, and excellent reliability, respectively^17^.

Accuracy tables and Bland–Altman plots were adapted based on the ESH-2010 protocol^18^.

Receiver operating characteristic (ROC) curves were constructed to assess the sensitivity and specificity of TestBP-V1.5 and to detect hypertension in comparison to RefBP.

The diagnostic performance of the TestBP-V1.5 was further analysed by calculating Youden’s index and defining highest value as optimal systolic and diastolic cut-off value for mean 24 h, awake, asleep BP for the TestBP compared to RefBP. Youden’s index (J) was calculated as (J) = sensitivity + specificity − 1.

Cut-off values for RefBP were based on the 2018 ESH/ESC Practice Guidelines for the Management of Arterial Hypertension using definitions of hypertension by office and out-of-office BP levels^3^.

Positive predictive value (PPV) and negative predictive value (NPV) of the TestBP measurements were calculated with sensitivity, specificity and the corresponding prevalence of elevated BP according to the RefBP measurements in our cohort of participants.

This study was designed and performed without patient involvement. Participants and or patients were not invited to remark on the study design and were not consulted to change patient relevant outcomes or interpret the results. Patients were not asked to contribute to the writing or editing of this manuscript for readability or accuracy.

Statistical analyses were performed using SAS version 9.4 (SAS Institute Inc, Cary, NC) and SPSS Version 22 (IBM), a p-value of < 0.05 was pre-specified to indicate statistical significance.

## Results

### Baseline characteristics

Consecutive enrolment of 83 individuals took place from May to December 2017. Twelve participants were excluded due to insufficient recording times, poor recording quality, technical problems, and atrial fibrillation^7^. Finally, a total of 71 participants’ data were analysed. Table 1 shows baseline characteristics. Mean systolic and diastolic BP (SD) of the calibration measurements were 139.5 (21.9) and 85.8 (15.1) mmHg, respectively.

**Table 1:** Baseline characteristics**.**

Characteristic	Overall (n = 71)
Sex (male), n	36 (50.7)
Age, years	49.3 (15.1, (25–82))
BMI, kg/m^2^	26.7 (5.4)
Mean valid 24 h cuff-based BP readings, n	59.6 (5.2, (37–66))
Antihypertensive treatment, n	28 (39.4)

Data are mean (± standard deviation, (range)) or counts (percentage), as appropriate.

*BP* blood pressure, *BMI* Body Mass Index.

### Comparison of the RefBP versus TestBP-V1.4 and TestBP-V1.5

Mean systolic and diastolic 24 h, awake and asleep values of RefBP, TestBP-V1.4 and TestBP-V1.5 are presented in Table 2 showing statistically significant differences in all comparisons—with TestBP-V1.5 being closer to RefBP than TestBP-V1.4 especially in asleep BP values.

**Table 2:** Comparison of systolic and diastolic blood pressure measurements measured by RefBP, TestBP-V1.4 and TestBP-V1.5.

	RefBP	TestBP-V1.4	TestBP-V1.5	p-values		
				RefBP vs. TestBP-V1.4	RefBP vs. TestB-V1.5	TestBP-V1.4 vs TestBP-V1.5
**Systolic**						
24 h	134.0 (17.3)	140.8 (20.0)	139.1 (20.1)	< 0.0001	0.0002	< 0.0001
Awake	138.1 (18.0)	142.0 (20.2)	142.3 (20.2)	0.002	0.001	0.02
Asleep	122.1 (18.4)	138.7 (20.1)	134.1 (20.5)	< 0.0001	< 0.0001	< 0.0001
**Diastolic**						
24 h	79.3 (11.7)	85.8 (14.1)	83.5 (13.0)	< 0.0001	< 0.0001	< 0.0001
Awake	82.8 (12.4)	86.7 (13.9)	86.2 (14.2)	< 0.0001	0.0002	0.003
Asleep	69.5 (11.2)	84.5 (14.6)	79.1 (14.6)	< 0.0001	< 0.0001	< 0.0001

Data are mean (± standard deviation), p-values are based on paired T-tests.

*BP* blood pressure. BP expressed as mmHg.

### Bland–Altman plots for comparison of the RefBP, TestBP-V1.4 and TestBP-V1.5

**Figure 1:**
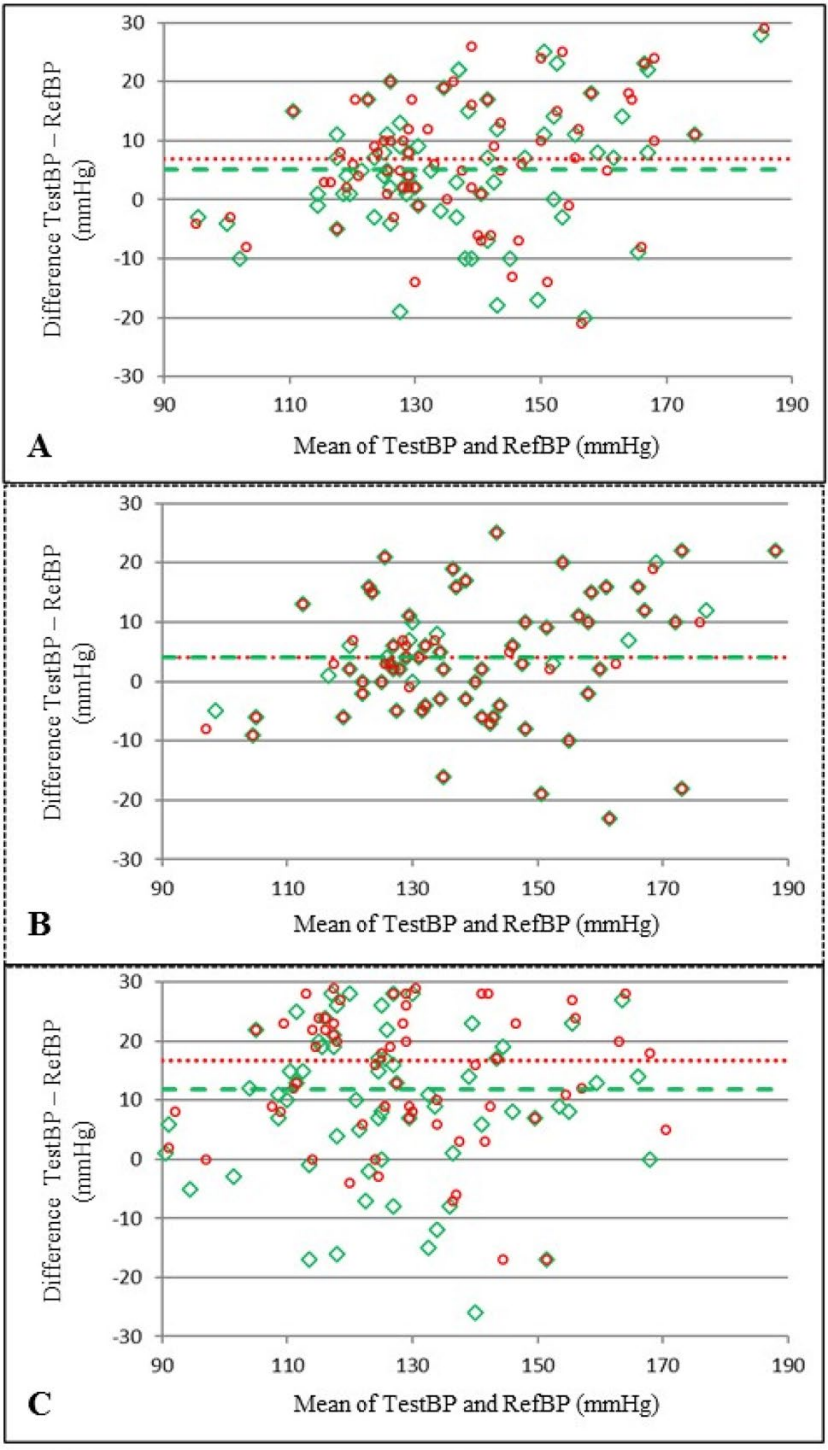
Bland Altman plots comparing systolic mean (**A**) 24 h, (**B**) awake and (**C**) asleep. BP values of TestBP-V1.4 /V1.5 and Ref BP and RefBP. Horizontal lines indicate mean differences Legend: Version 1.4—open red circle, Version 1.5—open green diamond, Version 1.4—red dotted line, and Version 1.5—green dashed line.

Bland–Altman plots comparing mean systolic, diastolic and asleep BP values of TestBP-V 1.4/V1.5 and RefBP are shown (Fig. 1), illustrating that the best agreements of the TestBP-V1.4 and V1.5 to RefBP were seen during the awake measurements. Of note, better agreements during asleep were measured with TestBP-V1.5. Overall BP values were systematically higher with both Test BP versions.

### Intraclass correlation coefficients of RefBP and TestBP-V1.4 and TestBP-V1.5

The intraclass correlation coefficients (ICC) for mean systolic and diastolic 24 h, awake and asleep BP are shown in Table 3. The ICC show a good to excellent range of conformity with the exception of systolic and diastolic asleep values (moderate reliability)^17^. With TestBP-V1.5, ICC were especially higher for systolic and diastolic asleep values but did not improve after the update for systolic and diastolic awake values.

**Table 3:** ICC between RefBP and TestBP-V1.4 and TestBP-V1.5.

*n* = 71	TestBP-V1.4	TestBP-V1.5
24 h systolic	0.88	0.89
Awake systolic	0.91	0.91
Asleep systolic	0.70	0.75
24 h diastolic	0.85	0.89
Awake diastolic	0.90	0.90
Asleep diastolic	0.63	0.72

Data are Intraclass Correlation Coefficiants (ICC). Values less than 0.5, between 0.5 and 0.75, between 0.75 and 0.9, and greater than 0.90 indicating poor, moderate, good, and excellent reliability, respectively^17^.

### Standard errors of the measurements between RefBP and TestBP-V1.4 and TestBP-V1.5

The greatest difference between RefBP and TestBP-V1.4 and TestBP-V1.5 was found in asleep systolic BP measurements. Standard error of measurements for TestBP-V1.4 for mean systolic and diastolic 24 h, awake and asleep BP values was 6.5, 5.7, and 11.4 mmHg and 5.1, 4.2 and 9.1 mmHg respectively. Standard error of measurements for TestBP-V1.5 for systolic and diastolic 24 h mean, awake and asleep BP values was 6.2, 5.7, and 10.1 mmHg and 4.4, 4.6 and 7.3 mmHg, respectively (Table 4).

**Table 4:** Standard error of the measurement between the TestBP-V1.4 and TestBP-V1.5 with the RefBP.

n = 71	TestBP-V1.4	TestBP-V1.5
24 h systolic	6.5 [12.7]	6.2 [12.2]
Awake systolic	5.7 [11.2]	5.7 [11.2]
Asleep systolic	11.4 [22.3]	10.1 [19.8]
24 h diastolic	5.1 [10.0]	4.4 [8.6]
Awake diastolic	4.2 [8.2]	4.2 [8.2]
Asleep diastolic	9.1 [17.8]	7.3 [14.3]

Data are standard error of the measurements [95% confidence interval].

### Agreement of measurements between the RefBP vs. TestBP-V1.5

Overall, when comparing mean 24 h systolic BPM of RefBP and TestBP-V1.5 agreements ≤ 2, ≤ 5, ≤ 10, and ≤ 15 mmHg were 16.9%, 38%, 62%, 77.5% respectively. In the awake intermediate BP group (135–150 mmHg), the best agreements were seen. The asleep BP groups had the poorest agreements (Table 5).

**Table 5:** Agreement between RefBP and TestBP-V1.5 for mean systolic (panel A) and diastolic (panel B) 24 h, awake and asleep blood pressure values, stratified by mean systolic daytime blood pressure and mean diastolic awake blood pressure respectively.

A. systolic values n (%)	< 135 mmHg low (n = 36)	≥ 135; < 150 mmHg intermediate (n = 15)	≥ 150 mmHg high (n = 20)	Overall (n = 71)
**24 h**				
Mean difference, mmHg	6.0 (8.1)	2.3 (11.5)	5.7 (14.6)	5.1 (10.9)
Mean absolute difference, mmHg				9.6 (7.2)
≤ 2	8 (22.2%)	3 (20.0%)	1 (5.0%)	12 (16.9%)
≤ 5	19 (52.8%)	5 (33.3%)	3 (15.0%)	27 (38.0%)
≤ 10	26 (72.2%)	10 (66.7%)	8 (40.0%)	44 (62.0%)
≤ 15	31 (86.1%)	12 (80.0%)	12 (60.0%)	55 (77.5%)
**Awake**				
Mean difference, mmHg	5.1 (8.4)	0.7 (8.7)	4.9 (13.9)	4.1 (10.3)
Mean absolute difference, mmHg				8.8 (6.9)
≤ 2	9 (25.0%)	2 (13.3%)	2 (10.0%)	13 (18.3%)
≤ 5	18 (50.0%)	6 (40.0%)	3 (15.0%)	27 (38.0%)
≤ 10	27 (75.0%)	13 (86.7%)	8 (40.0%)	48 (67.6%)
≤ 15	30 (83.3%)	13 (86.7%)	12 (60.0%)	55 (77.5%)
**Asleep**				
Mean difference, mmHg	12.1 (10.7)	10.8 (18.0)	12.5 (19.1)	11.9 (14.9)
Mean absolute difference, mmHg				15.8 (10.6)
≤ 2	4 (11.1%)	1 (6.7%)	1 (5.0%)	6 (8.5%)
≤ 5	8 (22.2%)	1 (6.7%)	1 (5.0%)	10 (14.1%)
≤ 10	14 (38.9%)	5 (33.3%)	7 (35.0%)	26 (36.6%)
≤ 15	22 (61.1%)	7 (46.7%)	10 (50.0%)	39 (54.9%)

B. diastolic values n (%)	< 85 mmHg low (n = 44)	≥ 85; < 95 mmHg intermediate (n = 16)	≥ 95 mmHg high (n = 11)	Overall (n = 71)
**24 h**				
Mean difference, mmHg	4.2 (6.5)	3.1 (8.2)	5.6 (9.9)	4.2 (7.4)
Mean absolute difference, mmHg				6.8 (5.1)
≤ 2	9 (20.5%)	5 (31.3%)	1 (9.1%)	15 (21.1%)
≤ 5	25 (56.8%)	6 (37.5%)	5 (45.5%)	36 (50.7%)
≤ 10	35 (79.5%)	11 (68.8%)	7 (63.6%)	53 (74.7%)
≤ 15	43 (97.7%)	16 (100%)	9 (81.8%)	68 (95.8%)
**Awake**				
Mean difference, mmHg	3.6 (6.2)	2.1 (7.7)	4.3 (11.0)	3.4 (7.4)
Mean absolute difference, mmHg				6.4 (5)
≤ 2 mmHg	13 (29.6%)	3 (18.8%)	2 (18.2%)	18 (25.4%)
≤ 5 mmHg	22 (50.0%)	9 (64.3%)	4 (36.4%)	35 (49.3%)
≤ 10 mmHg	39 (88.6%)	14 (87.5%)	7 (63.6%)	60 (84.5%)
≤ 15 mmHg	43 (97.7%)	15 (93.8%)	9 (81.8%)	67 (94.4%)
**Asleep**				
Mean difference, mmHg	8.6 (9.2)	10.3 (10.5)	13.0 (10.8)	9.7 (9.8)
Mean absolute difference, mmHg				11.7 (7.1)
≤ 2	5 (11.4%)	1 (6.3%)	0 (0%)	6 (8.5%)
≤ 5	13 (29.5%)	4 (25.0%)	1 (9.1%)	18 (25.4%)
≤ 10	24 (54.5%)	8 (50.0%)	4 (36.4%)	36 (50.7%)
≤ 15	32 (72.7%)	8 (50.0%)	6 (54.5%)	46 (64.8%)

Data are mean (± standard deviation) or counts (percent).

Overall, in the diastolic measurements, we saw the following agreements for ≤ 2, ≤ 5, ≤ 10, and ≤ 15 mmHg, 21.1%, 50.7%, 74.7% and 95.8% respectively. Best agreements were seen in the awake low and intermediate BP groups and the poorest agreements in the asleep BP values (Table 5).

### Diagnostic accuracy of TestBP-V1.5 for the detection of hypertension compared to gold standard diagnosis by RefBP

**Figure 2:**
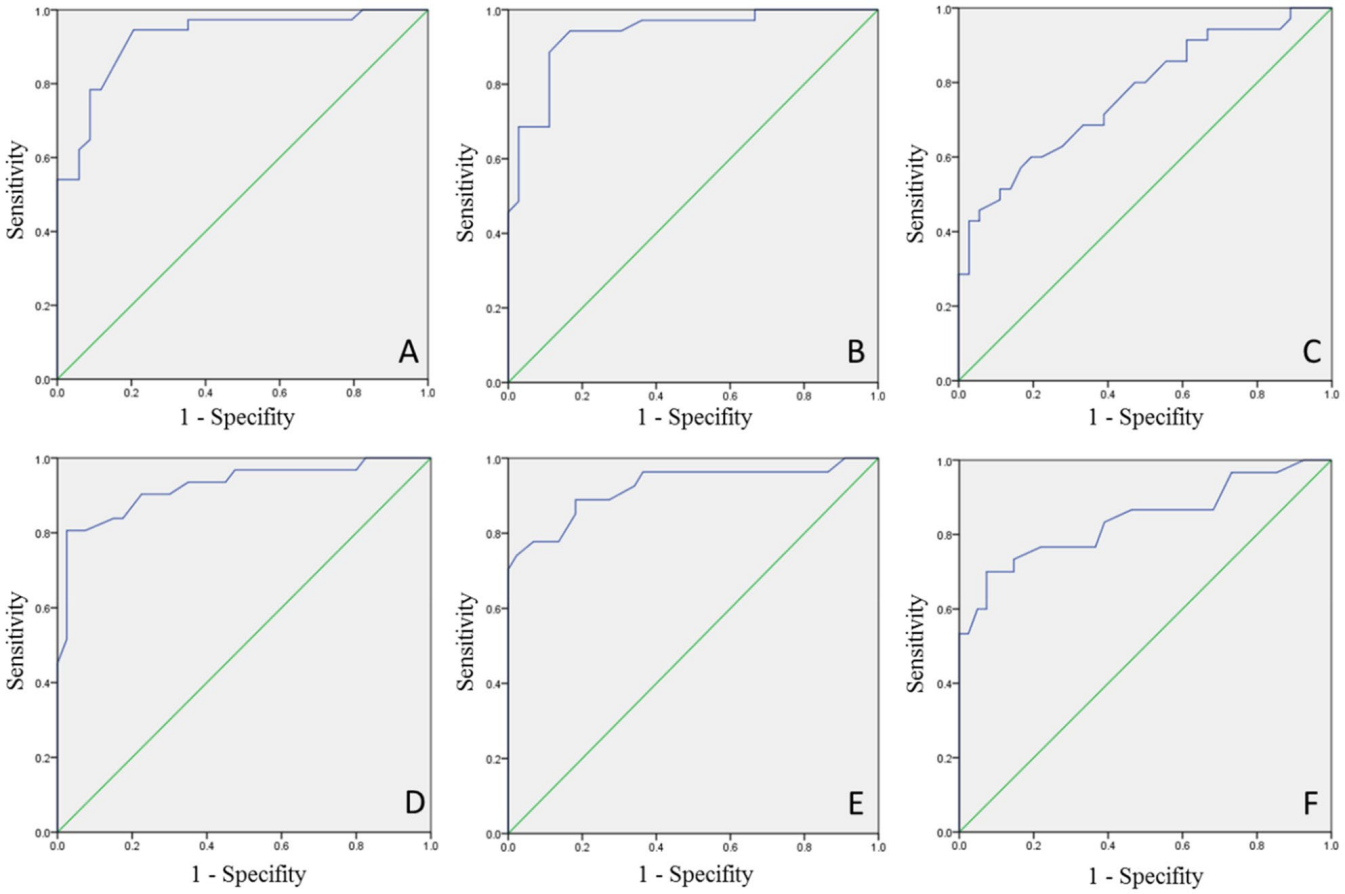
Receiver operating characteristic curve for systolic (**A**) 24 h, (**B**) awake, (**C**) asleep and diastolic, (**D**) 24 h, (**E**) awake and (**F**) asleep BP levels.

The area under the curve (AUC) (95% confidence interval) for the TestBP-V1.5 in detecting hypertension in comparison to the RefBP was 0.92 (0.86; 0.99), 0.94 (0.88; 0.99) and 0.77 (0.66; 0.88) for systolic 24 h, awake and asleep BP values and 0.92 (0.86; 0.99), 0.92 (0.85; 0.99) and 0.84 (0.74; 0.94) for diastolic 24 h, awake and asleep BP values, respectively (Fig. 2).

### Cut-off values for the clinical interpretation of the TestBP-V1.5

For the detection of hypertension, TestBP-V1.5 characteristics for standard mean systolic and diastolic 24 h cut-off values and for highest Youden’s indices are shown in Table 6. TestBP-V1.5 had 95% sensitivity, 65% specificity, a PPV of 74% and a NPV of 92% when applying a standard systolic diagnostic cut-off value of 130 mmHg, and a 90% sensitivity, 70% specificity, a 70% PPV and a 90% NPV when applying standard diastolic cut-off values of 80 mmHg. Highest Youden’s Index for mean systolic and diastolic 24 h BP was at systolic 133 mmHg (95% sensitivity, 80% specificity, 83% PPV, and 93% NPV) and diastolic 87 mmHg (70% sensitivity, 93% specificity, 87% PPV, and 81% NPV) respectively. Additional cut-off values (representing ≥ 95% sensitivity or ≥ 95% specifity) corresponding to TestBP-V1.5 awake and asleep systolic and diastolic values are show in Supplementary Table S1.

**Table 6:** Test characteristics for different TestBP-V1.5 values for systolic and diastolic mean 24 h, awake, and asleep.

Test characteristics of TestBP-V1.5at different cut-offs	Sensitivity (%)	Specificity (%)	PPV (%)	NPV (%)
Standard cut-off for the detection of systolic mean 24 h hypertension 130 mmHg	95	65	74	92
**Highest Youden’s Index for systolic mean 24 h hypertension at 133 mmHg**	95	80	83	93
Standard cut-off for the detection of diastolic mean 24 h hypertension 80 mmHg	90	70	70	90
**Highest Youden’s Index for diastolic mean 24 h hypertension at 87 mmHg**	81	98	96	87
Standard cut-off for the detection of mean systolic awake hypertension 135 mmHg	94	78	80	93
**Highest Youden’s Index for systolic mean awake hypertension at 135 mmHg**	94	78	80	93
Standard cut-off for the detection of mean diastolic awake hypertension 85 mmHg	89	75	68	92
**Highest Youden’s Index for diastolic mean awake hypertension at 91 mmHg**	74	98	95	86
Standard cut-off for the detection of mean systolic asleep hypertension 120 mmHg	91	33	57	80
**Highest Youden’s Index for systolic asleep hypertension at 136 mmHg**	60	80	75	68
Standard cut-off for the detection of mean asleep diastolic hypertension 70 mmHg	87	39	51	80
**Highest Youden’s Index for diastolic asleep hypertension at 83 mmHg**	70	93	87	81

Hypertension determined by RefBP with standard cut-offs (systolic/diastolic 24mean 130/80 mmHg, awake 135/85 mmHg, asleep 120/70 mmHg)^2^.

## Discussion

Over a century, non-invasive cuff-based BP measurement, using the initial approach of Scipione Riva Rocci and Nicolai Sergeivich Korotkoff remained more or less unchanged until the advances through the development of oscillometric devices in the last decades^19^. Recently, devices using different techniques such as PTT or pulse wave analysis have increased, challenging the clinician to understand these new measurements. Generally guidelines suggest the use of validated devices in clinical practice however some newer techniques fail validation when compared to standard measurements^2^. A caveat in the validation of BP devices may be that the ESH-IP 2010 protocol was developed to compare single device measurements to single gold standard measurements using a mercury sphygmomanometer under laboratory conditions. This is useful and adequate to validate standard cuff based BP devices, but may give a misleading security for 24 h BP measurement devices with alternative techniques and beat-to-beat BP determination^18,20^. Therefore in 2017 our group evaluated the accuracy of a ESH-IP 2010 validated cuffless PTT device (Test BP) against a cuff-based standard device (Ref BP) over 24 h under usual clinical condition^7^.

We showed a significant difference in all measurements (mean 24 h, awake, and asleep), with the most divergence seen during assessment of blood pressure during sleep. The TestBP revealed higher BP values than the RefBP and overall, the study brought attention to the fact that, the two devices which measure BP differently came out with significant differences in values^7^, Highlighting that results of cuff-less and cuff-based devices are not directly interchangeable in clinical practice.

In July 2018, the TestBP device’s manufacturers updated the software algorithm. This created the need to reassess the data and evaluate the diagnostic accuracy of the TestBP device. Comparing the RefBP to the TestBP-V1.4 and TestBP-V1.5, we see improvements in the accuracy of measurements in relation to the RefBP especially for the asleep diastolic values with various modes of comparison. However, significantly higher measurements are still seen with the TestBP device, as was apparent before the update. Improvement in data output in terms of better agreement to RefBP was seen overall but especially with asleep values. Best agreement was in the high normal to grade 1-hypertension categories.

Therefore, the issue remains that measurements of the RefBP and the TestBP are not interchangeable. The fact that the device is on the market and already in clinical use, led to the further analysis of how the TestBP-V1.5 results could reliably discriminate between normotension and hypertension. In doing, a compass for clinical interpretation could be created with the aim of device specific cut-off values. Therefore, we used an alternative approach to analyze the data, implementing a technique used when determining the diagnostic accuracy of biomarkers against a gold standard diagnosis. In our case, the TestBP device was the biomarker and the RefBP was the gold standard to create the adjudicated diagnosis of hypertension according to standard cut-off values given in the 2018 ESC/ESH guidelines^2^. Our ROCs demonstrated that for systolic and diastolic mean 24 h and awake measurements, the TestBP-V1.5 has a high diagnostic accuracy with an AUC of ≥ 0.92–0.94 while the TestBP-V1.5 showed less ability to accurately detect systolic and diastolic hypertension during asleep measurements with an AUC of 0.77 and 0.84, respectively when compared to the RefBP. Using Youden’s index as the optimal device specific cut-off value to detect hypertension, we found cut-off values of 133/87 mmHg for 24 h mean, 136/91 mmHg awake and 136/83 mmHg asleep best for the distinction of hypertension and normotension.

With the awareness that the TestBP derives higher BP values than the RefBP, the calculated PPV, NPV, and Youden’s Index may help to guide clinicians on how to interpret the results of the TestBP. This is a vital step when incorporating new devices into practice—especially when usual validation protocols were limited due to the measurement technique.

### Strengths and limitations

The strength of our study was that our data reflected a 24 h period, simultaneously created with two different devices. To date, this is the largest cohort comparing the TestBP device to a RefBP device simultaneously over a 24 h period in a routine clinical setting. Since this was an investigator-initiated trial with the aim of evaluating the accuracy of the PTT device in clinical practice, we had no insights into the technical changes applied by the manufacturer to this latest algorithm update. This software update is now available and in use by customers. Therefore our study adds very important information for these practitioners.

This study implemented the widely used Spacelabs 90217A 24 h BPM device as a clinically validated reference device^11^. All cuff-based BPM devices show differences when compared to the gold standard for device validation, which are comparisons to mercury sphygmomanometers, the reported mean differences and accuracy tables may differ slightly in a comparison to another cuff-based 24 h measurement device. Nevertheless, the Spacelabs device meets the Association for the Advancement of Medical Instrumentation’s standard for standing, sitting and supine measurements and was graded “A” for systolic and diastolic BP in a modified British Hypertension Society protocol.

As mentioned in the methods section of the paper we used 27 measurements as the minimum number for a valid RefBP measurement set. This criteria is in line with current recommendations in clinical settings. Although in some research settings stricter criteria are sometimes applied, we would like to emphasize that this study focused on the comparability of two devices in a typical clinical setting and even then that we maintained a very high quality control standard with an average of 60 measurements per participant (Table 1).

Another limitation is for the derivation of device specific cut-off values, more participants should be enrolled from various demographics (age, race, comorbidities, BP ranges). Therefore, the current data should be interpreted in the framework of a pilot evaluation.

## Conclusions

We showed that the software update significantly improved the agreement of the tested cuff-less device (TestBP) in comparison to the cuff-based device (RefBP). This development emphasized that asleep values improved with the software update. In summary, we showed that TestBP-V1.5 correlates better to the RefBP than TestBP-V1.4. After the software update, the difference between the RefBP and the TestBP-V1.5 is less but remains statistically and clinically significant. When applying device specific cut-off points the TestBP device may be a reliable tool for the detection of systolic and diastolic hypertension for 24 h and awake BP values, but less so for asleep measurements.

## Supplementary Information

Supplementary Information.
