# Supplementary material for: Improved agreement and diagnostic accuracy of a cuffless 24-h blood pressure measurement device in clinical practice
Source: Sci Rep. 2021 Jan 13;11:1143. doi: 10.1038/s41598-020-80905-x (PMC7806663; doi:10.1038/s41598-020-80905-x)
Supplement: Supplementary file 1 — Supplementary Information. [file 41598_2020_80905_MOESM1_ESM.docx]

**Supplementary Table 1. Additional cut-off values (representing ≥95% sensitivity or ≥95% specifity) corresponding to TestBP-V1.5 awake and asleep systolic and diastolic values.**

| **Additional Test Characteristics of TestBP-V1.5** |  | **Sensitivity** | **Specificity** | **PPV** | **NPV** |
| --- | --- | --- | --- | --- | --- |
|  | **Hypertension by RefBP, systolic mean 24h BP ≥130mmHg** | | | | |
| ≥95% sensitivity | 133 mmHg | 95% | 80% | 83% | 93% |
| ≥95% specifity | 148 mmHg | 54% | 100% | 100% | 67% |
|  | **Hypertension by RefBP, diastolic mean 24 BP ≥ 80mmHg** | | | | |
| ≥95% sensitivity | 77 mmHg | 97% | 53% | 62% | 95% |
| ≥95% specifity | 87 mmHg | 81% | 98% | 96% | 87% |
|  | **Hypertension by RefBP, systolic mean awake BP ≥ 135mmHg** | | | | |
| ≥95% sensitivity | 132mmHg | 97% | 64% | 72% | 96% |
| ≥95% specifity | 148 mmHg | 67% | 97% | 96% | 76% |
|  | **Hypertension by RefBP, diastolic mean awake BP ≥ 85 mmHg** | | | | |
| ≥95% sensitivity | 83 mmHg | 96% | 64% | 62% | 96% |
| ≥95% specifity | 92 mmHg | 74% | 98% | 95% | 86% |
|  | **Hypertension by RefBP, systolic mean asleep BP ≥ 120 mmHg** | | | | |
| ≥95% sensitivity | 107 mmHg | 97% | 11% | 51% | 80% |
| ≥95% specifity | 149 mmHg | 43% | 97% | 94% | 64% |
|  | **Hypertension by RefBP, diastolic mean asleep BP ≥ 70 mmHg** | | | | |
| ≥95% sensitivity | 65 mmHg | 97% | 27% | 49% | 92% |
| ≥95% specifity | 86 mmHg | 60% | 95% | 90% | 77% |
